# Areas of improvement in the medical care of SMA: evidence from a nationwide patient registry in Germany

**DOI:** 10.1186/s13023-023-02639-z

**Published:** 2023-02-21

**Authors:** Berenike Leibrock, Erik Landfeldt, Justine Hussong, Tabea Huelle, Hannah Mattheus, Simone Thiele, Maggie C. Walter, Michael Zemlin, Eva Moehler, Ullrich Dillman, Sophia Abner, Marina Flotats-Bastardas

**Affiliations:** 1 University of Saarland, Saarbruecken, Germany; 2 IQVIA, Stockholm, Sweden; 3 Department of Child and Adolescent Psychiatry, Saarland University Hospital, Homburg, Germany; 4 Department of Neurology, Friedrich-Baur-Institute, Ludwig-Maximilians-University of Munich, Munich, Germany; 5 Department of General Pediatrics and Neonatology, Saarland University Hospital, Homburg, Germany; 6 Department of Neurology, Saarland University, Homburg/Saar, Germany; 7 IQVIA, London, UK; 8 Department of Neuropaediatrics, Saarland University Hospital, Homburg, Germany

**Keywords:** (8/10): spinal muscular atrophy, Nusinersen, Risdiplam, Medical care, Rehabilitation, Non-invasive ventilation, Motor function, Bulbar function

## Abstract

**Background:**

Management and treatment of spinal muscular atrophy (SMA) has changed in recent years due to the introduction of novel transformative and potentially curative therapies resulting in the emergence of new disease phenotypes. Yet, little is known about the uptake and impact of these therapies in real-world clinical practice. The objective of this study was to describe current motor function, need of assistive devices, and therapeutic and supportive interventions provided by the healthcare system, as well as the socioeconomic situation of children and adults with different SMA phenotypes in Germany. We conducted a cross-sectional, observational study of German patients with genetically confirmed SMA identified and recruited via a nationwide SMA patient registry (www.sma-register.de) within the TREAT-NMD network. Study data was recorded directly from patient-caregiver pairs through a study questionnaire administered online via a dedicated study website.

**Results:**

The final study cohort consisted of 107 patients with SMA. Of these, 24 were children and 83 adults. In total, about 78% of all participants were taking medication for SMA (predominantly nusinersen and risdiplam). All children with SMA1 were able to sit and 27% of children with SMA2 were able to stand or walk. Impaired upper limb function, scoliosis and bulbar dysfunction were observed more frequently in patients with reduced lower limb performance. Physiotherapy, occupational therapy, and speech therapy, as well as the use of cough assists were less common than indicated by care guidelines. Family planning and educational and employment status appear to be related to motor skill impairment.

**Conclusions:**

We show that the natural history of disease has changed in Germany following improvements in SMA care and the introduction of novel therapies. Yet, a non-trivial proportion of patients remain untreated. We also identified considerable limitations in rehabilitation and respiratory care, as well as low labour-market participation among adults with SMA, calling for action to improve the current situation.

**Supplementary Information:**

The online version contains supplementary material available at 10.1186/s13023-023-02639-z.

## Background

Spinal muscular atrophy (SMA) is a genetic neurodegenerative disease of the second motoneuron that causes progressive impairment of motor skills. Inheritance of SMA is autosomal recessive due to mutations of the survival motor neuron (*SMN1*) gene. Yet, depending on the expression of the paralougous *SMN2* gene, there is considerable variability in disease onset, morbidity, and life expectancy [1, 2].

SMA is traditionally classified into four subtypes according to age at onset of clinical symptoms and highest motor function milestone achieved. In SMA type 1 (SMA1), disease onset occurs in the first six months of life with impaired head control and progressive weakness of the respiratory muscles, so that most children require invasive ventilation or die before 2 years of age. In SMA type 2 (SMA2), the disease begins before 18 months of age, children show motor developmental delay, with sitting being the highest motor milestone achieved, which some lose over time into adulthood. In SMA type 3 (SMA3), all patients are able to walk, and two subtypes are distinguished: type 3a, with disease onset before 3 years of age and tendency to lose the ability to walk in adulthood, and type 3b, with disease onset beyond 3 years of age [2]. Until now, adult SMA frequently included patients who had previously been classified as SMA4, since they only were diagnosed in adulthood when symptoms of motor involvement became more disabling. However, careful exploration of the medical history often point towards an earlier onset in late childhood or adolescence, and the majority of patients also harbor 3–4 SMN2 copies, same as seen in patients with SMA3, so the existence and frequency of a SMA type 4 (SMA4) phenotype is controversial [3].

In recent years, the introduction of several novel treatments that target *SMN2* splicing to increase SMN protein expression (e.g., nusinersen and risdiplam) has dramatically transformed the prognosis of SMA [4, 5]. As a result, it is therefore now possible to modify the natural history of the disease, resulting in new phenotypes that are challenging to classify [6]. However, in most geographical areas, including Germany, little is known about the uptake and impact of these therapies in real-world clinical practice [7–10].

The objective of this study was to describe current motor function, need of assistive devices, and therapeutic and supportive interventions provided by the healthcare system, as well as the socioeconomic situation of children and adults with different SMA phenotypes. Our aim was to identify areas of improvement in the current medical management of SMA in Germany.

## Results

### Subtypes and diagnosis

Out of 742 patients enrolled in the SMA patient registry, 513 met the inclusion criteria, and 117 agreed to participate in the study. Complete data was available for 24 children and 83 adults with SMA (Table 1). The largest subgroups consisted of adults with SMA2 and 3. Only one participant was diagnosed with SMA4. In consideration of current motor function, the largest subgroup consisted of adult sitters.

**Table 1:** Demographic characteristics of the patient sample, by SMA type and by current motor function

	Total sample, by SMA subtype (n = 107)									Total sample, by current motor function (n = 107)						Total
	SMA1		SMA2		SMA3a		SMA3b		SMA4	Non-sitter		Sitter		Walker		
	Child	Adult	Child	Adult	Child	Adult	Child	Adult	Adult	Child	Adult	Child	Adult	Child	Adult	
	(n = 5)	(n = 3)	(n = 11)	(n = 29)	(n = 5)	(n = 14)	(n = 3)	(n = 36)	(n = 1)	(n = 4)	(n = 7)	(n = 13)	(n = 56)	(n = 7)	(n = 20)	(n = 107)
Mean age, in years (SD)	7.4 (4.3)	31.3 (18.1)	10.6 (4.9)	32.1 (9.2)	9.6 (4.9)	51.2 (12.5)	11.3 (3.2)	47.7 (15.1)	48 (-)	13 (3.5)	39 (15.5)	9.4 (5.0)	41.3 (16.0)	8.9 (3.8)	46.2 (12.0)	35 (19.1)
Female sex, n (%)	5 (100)	2 (67)	5 (45)	19 (66)	3 (60)	8 (57)	–	14 (39)	–	2 (50)	4 (57)	9 (69)	31 (55)	2 (29)	8 (40)	56 (52)
Living situation*																
Alone, n (%)	–	–	–	8 (28)	–	3 (21)	–	4 (11)	–	–	1 (14)	–	13 (23)	–	1 (5)	15 (14)
With own family, n (%)	–	–	–	8 (28)	–	10 (71)	–	24 (67)	1 (100)	–	3 (43)	–	22 (39)	–	18 (90)	43 (40)
With parents, n (%)	–	2 (67)	–	10 (35)	–	1 (7)	–	5 (14)	–	–	3 (43)	–	15 (27)	–	–	18 (17)
Other, n (%)	–	1 (33)	–	3 (10)	–	–	–	3 (8)	–	–	–	–	6 (11)	–	1 (5)	7 (7)
Married or in a relationship, n (%)**	4 (80)	–	11 (100)	14 (48)	5 (100)	11 (79)	3 (100)	28 (78)	1 (100)	4 (100)	4 (57)	12 (92)	30 (54)	7 (100)	20 (100)	77 (72)
*Education (highest completed)*																
Pre–school/ no school, n (%)	3 (60)	–	3 (27)	–	2 (40)	–	–	–	–	–	–	5 (39)	–	3 (43)	–	8 (8)
Primary school, n (%)	1 (20)	1 (33)	2 (18)	1 (3)	1 (20)	2 (14)	2 (67)	4 (11)	–	1 (25)	2 (29)	3 (23)	6 (11)	2 (29)	–	14 (13)
Secondary school, n (%)	1 (20)	1 (33)	3 (27)	4 (14)	1 (20)	5 (36)	1 (33)	11 (31)	1 (100)	2 (50)	–	3 (23)	13 (23)	1 (14)	9 (45)	28 (26)
High school/ general certificate, n (%)	–	1 (33)	3 (27)	24 (83)	1 (20)	7 (50)	–	21 (58)	–	1 (25)	5 (71)	2 (15)	37 (66)	1 (14)	11 (55)	57 (53)
Highest vocational training*																
None, n (%)	–	1 (33)	–	8 (28)	–	–	–	2 (6)	–	–	2 (29)	–	8 (14)	–	1 (5)	11 (10)
Apprenticeship, n (%)	–	1 (33)	–	9 (31)	–	7 (50)	–	24 (66)	1 (100)	–	3 (43)	–	27 (48)	–	12 (60)	42 (39)
University studies, n (%)	–	1 (33)	–	12 (41)	–	7 (50)	–	10 (28)	–	–	2 (29)	–	21 (38)	–	7 (35)	30 (28)
Currently employed, n (%)**	5 (100)	2 (67)	11 (100)	18 (62)	5 (100)	9 (64)	3 (100)	20 (55)	–	4 (100)	4 (57)	13 (100)	31 (55)	7 (100)	14 (70)	73 (68)

*Only adults

**For children: referring to the parents

### Socioeconomic characteristics and medical care

Table 2 shows socioeconomic and medical care data of the study population. All adult walkers were in a relationship and most of them lived with their own family, in contrast to non-sitters and sitters (*p* < 0.001 and *p* = 0.007, respectively). Overall, nearly 60% of adult patients had the two highest German school-leaving qualifications that qualify for university studies (i.e., *Abitur* or *Fachabitur*) and 36% had a university degree. A significantly larger proportion of adults with SMA3 had completed vocational training (i.e., had learned a profession) compared to adults with SMA2 (*p* = 0.007). Looking at the sample by current motor function, 14% sitters and 5% walkers had no vocational training (*p* = 0.566). Across all SMA types, adult employment was around 60%. Patients in the non-sitter and sitter subgroups were employed in over 55% of cases and walkers in 70% (*p* = 0.517). In parents of children with SMA1, there was a tendency for only one parent to be employed compared to parents of other SMA types (*p* = 0.094).

**Table 2:** Clinical characteristics, functional ability, and medical care of patients with SMA, by SMA type and by current motor function

	Total sample, by SMA subtype (n = 107)									Total sample, by current motor function (n = 107)						Total
	SMA1		SMA2		SMA3a		SMA3b		SMA4	Non-sitter		Sitter		Walker		
	Child	Adult	Child	Adult	Child	Adult	Child	Adult	Adult	Child	Adult	Child	Adult	Child	Adult	
	(n = 5)	(n = 3)	(n = 11)	(n = 29)	(n = 5)	(n = 14)	(n = 3)	(n = 36)	(n = 1)	(n = 4)	(n = 7)	(n = 13)	(n = 56)	(n = 7)	(n = 20)	(n = 107)
Mean age at first symptoms, years (SD)	0 (0)	0.3 (0.6)	0.8 (0.8)	1.0 (0.6)	1.4 (0.6)	1.6 (0.5)	3.0 (0)	10.6 (8)	35 (0)	1 (0.8)	3 (5.4)	0.5 (0.8)	3.9 (6.8)	2 (1.0)	11.5 (8.7)	4.6 (7.2)
Mean age at SMA diagnosis, years (SD)	0.4 (0.5)	0.7 (0.6)	0.9 (0.7)	2.3 (3.2)	2.2 (0.5)	10.9 (13.1)	5.3 (0.6)	18.6 (12.9)	47 (0)	1 (0.8)	4.7 (6.5)	0.9 (1.0)	9 (12.6)	3.3 (2.0)	20.1 (13.0)	9.1 (12.3)
Duration of illness, years (SD)	7.4 (4.3)	31.0 (17.6)	9.8 (5.1)	31.2 (9.4)	8.2 (4.8)	49.6 (12.3)	9.1 (4.9)	37.1 (16)	13 (0)	12 (3.7)	36 (11.4)	8.9 (5.0)	37.4 (15.5)	6.9 (3.4)	34.7 (14.0)	30.4 (17.6)
*SMA type*																
SMA1, n (%)										–	1 (14)	5 (39)	2 (4)	–	–	8 (8)
SMA2, n (%)										4 (100)	4 (57)	6 (46)	25 (45)	1 (14)	–	40 (37)
SMA3, n (%)										–	2 (29)	2 (15)	29 (52)	6 (86)	19 (95)	58 (54)
SMA4, n (%)										–	–	–	–	–	1 (5)	1 (1)
*Best motor skills of lower extremities*																
None, n (%)	–	1 (33)	4 (36)	4 (14)	1 (20)	–	–	2 (6)	–							12 (11)
Sitting, n (%)	5 (100)	2 (67)	4 (36)	25 (86)	1 (20)	12 (86)	–	12 (33)	–							61 (57)
Standing, n (%)	–	–	2 (18)	–	–	–	–	5 (14)	–							7 (7)
Walking, n (%)	–	–	1 (9)	–	3 (60)	2 (14)	3 (100)	17 (47)	1 (100)							27 (25)
*Best motor skills of upper extremities*																
None, n (%)	1 (20)	2 (67)	–	4 (14)	–	1 (7)	–	–	–	–	1 (14)	1 (8)	6 (11)	–	–	8 (8)
Holding/picking up pen/coin with fingers, n (%)	1 (20)	–	–	10 (35)	–	2 (14)	–	5 (14)	–	–	3 (43)	1 (8)	12 (21)	–	2 (10)	18 (17)
Lifting hand to mouth, n (%)	3 (60)	1 (33)	7 (64)	15 (52)	1 (20)	5 (36)	–	8 (22)	1 (100)	4 (100)	3 (43)	7 (54)	24 (43)	–	3 (15)	41 (38)
Lifting hand above head, n (%)	–	–	4 (36)	–	4 (80)	6 (43)	3 (100)	23 (64)	–	–	–	4 (31)	14 (25)	7 (100)	15 (75)	40 (37)
Use of medical equipment for motor skills*																
None, n (%)	–	–	–	–	–	–	2 (67)	11 (31)	–	–	–	–	2 (4)	2 (29)	9 (45)	13 (12)
Orthoses, n (%)	5 (100)	2 (67)	6 (55)	8 (28)	2 (40)	–	–	2 (6)	–	1 (25)	1 (14)	10 (77)	9 (16)	2 (29)	2 (10)	25 (23)
Wheeled walker/ Crutches, n (%)	–	–	1 (9)	–	2 (40)	2 (14)	–	6 (17)	–	–	–	2 (15)	1 (2)	1 (14)	7 (35)	11 (10)
Wheelchair, n (%)	4 (80)	3 (100)	10 (91)	29 (100)	4 (80)	13 (93)	1 (33)	23 (64)	1 (100)	4 (100)	7 (100)	12 (92)	54 (96)	3 (43)	8 (40)	88 (82)
Scoliosis, n (%)	5 (100)	3 (100)	11 (100)	28 (97)	3 (60)	8 (57)	1 (33)	14 (39)	–	4 (100)	6 (86)	13 (100)	41 (73)	3 (43)	6 (30)	73 (68)
Operation of scoliosis, n (%)	1 (20)	–	7 (64)	20 (69)	2 (40)	1 (7)	–	3 (8)	–	4 (100)	2 (29)	6 (46)	22 (39)	–	–	34 (32)
*Food intake support*																
None, n (%)	1 (20)	–	8 (73)	10 (35)	5 (100)	12 (86)	3 (100)	34 (94)	1 (100)	2 (50)	3 (43)	8 (62)	34 (61)	7 (100)	20 (100)	74 (69)
None, but can eat only soft/puréed food, n (%)	–	2 (67)	–	5 (17)	–	–	–	–	–	–	1 (14)	–	6 (11)	–	–	7 (7)
G-tube, n (%)	3 (60)	–	1 (9)	5 (17)	–	–	–	–	–	–	2 (29)	4 (31)	3 (5)	–	–	9 (8)
Other, n (%)	1 (20)	1 (33)	2 (18)	9 (31)	–	2 (14)	–	2 (6)	–	2 (50)	1 (14)	1 (8)	13 (23)	–	–	17 (16)
*Ventilatory support*																
None, n (%)	–	1 (33)	5 (46)	11 (38)	5 (100)	11 (79)	3 (100)	33 (92)	1 (100)	–	2 (29)	6 (46)	36 (64)	7 (100)	19 (95)	70 (65)
Cough assist, n (%)	–	–	2 (18)	6 (21)	–	2 (14)	–	–	–	2 (50)	–	–	7 (13)	–	1 (5)	10 (9)
Non-invasive breathing support, n (%)	3 (60)	2 (67)	4 (36)	11 (38)	–	–	–	2 (6)	–	2 (50)	4 (57)	5 (39)	11 (20)	–	–	22 (21)
Invasive breathing support, n (%)	1 (20)	–	–		–	–	–	–	–	–	–	1 (8)	–	–	–	1 (1)
Other, n (%)	1 (20)	–	–	1 (3)	–	1 (7)	–	1 (3)	–	–	1 (14)	1 (8)	2 (4)	–	–	4 (4)
Supportive therapy*																
None, n (%)	–	1 (33)	–	4 (14)	–	–	–	9 (25)	–	–	1 (14)	–	9 (16)	–	4 (20)	14 (13)
Physiotherapy, n (%)	5 (100)	2 (67)	11 (100)	25 (86)	5 (100)	14 (100)	3 (100)	26 (72)	1 (100)	4 (100)	6 (86)	13 (100)	47 (84)	7 (100)	15 (75)	92 (86)
Occupational therapy, n (%)	2 (40)	–	3 (27)	4 (14)	1 (20)	2 (14)	–	3 (8)	1 (100)	1 (25)	–	3 (23)	8 (14)	2 (29)	2 (10)	16 (15)
Speech therapy, n (%)	4 (80)	1 (33)	1 (9)	9 (31)	1 (20)	–	1 (33)	1 (3)	–	–	1 (14)	5 (39)	9 (16)	2 (29)	1 (5)	18 (17)
Other, n (%)	2 (40)	–	1 (9)	1 (4)	1 (20)	2 (14)	–	7 (19)	–	–	–	3 (23)	6 (11)	2 (29)	4 (20)	15 (14)
*SMA-specific medication*																
None, n (%)	1 (20)	–	–	7 (24)	–	3 (21)	1 (33)	12 (33)	–	–	1 (14)	1 (8)	19 (34)	1 (14)	2 (10)	24 (22)
Nursinersen, n (%)	4 (80)	1 (33)	8 (73)	8 (28)	3 (60)	10 (71)	2 (67)	22 (61)	1 (100)	1 (25)	–	12 (92)	24 (43)	4 (57)	18 (90)	59 (55)
Risdiplam, n (%)	–	2 (67)	3 (27)	14 (48)	2 (40)	1 (7)	–	2 (6)	–	3 (75)	6 (86)	–	13 (23)	2 (29)	–	24 (22)

*Multiple answers possible

At the time of the survey, 78% of all participants were taking specific medication for SMA, most of them nusinersen, which had been given for a mean duration of 3 years. Adults with SMA2 mainly received risdiplam. In adults with SMA3, 30% of participants were not treated. Almost all children (92%) underwent SMA-specific medication, predominantly nusinersen. Looking at the sample by current motor function, almost all adult non-sitters (86%) were taking risdiplam and almost all walkers (90%) were receiving therapy with nusinersen. In the sitter group, 34% of adults received no medical therapy and those treated mainly took nusinersen.

### Lower limb motor function

Among adults with SMA2, 14% had lost their ability to sit. The majority of adults with SMA3a and 3b had lost their ability to walk (86% and 53%, respectively). By current motor function, the proportion of patients relying on assistive devices decreased from non-sitter to walker, but 40% of walkers used wheelchairs. All children with SMA1 were able to sit and two children with SMA2 had met more advanced milestones than sitting. Scoliosis was reported by 68% of the total cohort and its prevalence decreased with improvement in lower limb function.

### Upper limb motor function

The best upper limb function for 52% of adults with SMA2 was to raise their hands to their mouth and 14% of them received occupational therapy. In adults with SMA3, 16% could not raise their hand to mouth and 10% had occupational therapy. Looking at the sample according to current functional status, the prevalence of impairment in upper limb function decreased with improvement in lower limb function.

### Bulbar function

Adults with SMA2 needed significantly more support with food intake than adults with SMA3 (*p* < 0.001) and 31% of them were undergoing speech therapy. Looking at the adult sample by actual motor function ability, 57% of non-sitters and 39% of sitters needed assistance with food intake (i.e., soft food, G-Tube).

Overall, 35% of all patients required breathing support predominantly non-invasive ventilation (NIV). Among adults with SMA2, 21% used a cough assist. All children with SMA1 and 18% of children with SMA2 needed ventilatory support. Prevalence of respiratory support decreased with improved lower limb motor function.

We found a negative correlation between motor function and the prevalence of scoliosis (r =  − 0.408, *p* < 0.001) and the need for support for feeding (r =  − 0.358, *p* < 0.001) and a positive correlation between upper and lower limb motor function (r = 0.554, *p* < 0.001).

## Discussion

In this study, we examined demographic, clinical, and socioeconomic characteristics, as well as therapeutic and supportive interventions, among German children and adults with SMA identified via a nationwide disease-specific registry. Our findings reveal a change in the natural history of the disease, where all children with SMA1 acquired the ability to sit, and one child with SMA2 was able to walk. Natural history reports on SMA1 reveal that patients never achieve the ability to sit independently, and typically die because of respiratory failure at a median age of between 7 and 13 months [11, 12]. For patients with SMA2, natural history reports show that children achieve the ability to sit independently; yet, without specific therapy they never will be able to walk and over time some of them may lose the ability to sit independently [11, 13]. At the same time, our results are also indicative of the detrimental, progressive nature of SMA, where the majority of adults with SMA3 already had lost their ability to walk. Taking into account the age of our study population, this finding is in line with natural history reports [11, 13, 14]. We also found that impairment in upper limb function, scoliosis, and bulbar dysfunction were more common in patients with poor lower limb and trunk function, which again supports previous observations [15, 16]. Given the availability of novel, highly efficacious, and potentially curative therapeutic options and newborn screening programs ensuring timely identification and intervention, there is undoubtedly the potential for major phenotype shifts within the traditional SMA subtypes. The approach of classifying SMA according to the patient’s current level of motor function (i.e., non-sitter, sitter, walker) can thus help the medical community to monitor disease progress more accurately and to inform and improve appropriate medical care.

In total, 55% of all participants in this study were treated with nusinersen. This was in line with our expectations, as nusinersen was the first disease-specific treatment marketed in Germany for SMA (made available in 2017). However, a change in treatment preference in adults towards risdiplam was observed in those with decreasing lower limb motor function and higher scoliosis prevalence. Nevertheless, 22% of all participants still did not receive any specific therapy. In particular, this was most common among patients with SMA3 and sitters. Accordingly, there still seems to be some hesitation in, or barriers to, initiating specific treatment in this patient population in Germany. Possible explanations include lack of awareness of existing therapy options, disadvantages due to intrathecal application, and/or possible side-effects, especially in patients with slow disease progress or severe scoliosis [14, 17, 18].

We also identified notable deficiencies in the rehabilitation treatment of patients with SMA. For example, less than 25% of our cohort reported using orthoses and physiotherapy support among adults with SMA3b or walkers was also relatively infrequent. Indeed, orthoses can be helpful in SMA patients with impaired walking ability, regardless of motor subgroups, to support lower and upper limb function, and physiotherapy is recommended not only in children to improve motor development, but also in the adult population to promote function and mobility, as well as aerobic and general conditioning training [19]. Although impaired upper limb function is a potential limitation in everyday life, it is surprising that only a minority of participants receive functional rehabilitation treatment to improve hand skills. Therefore, awareness should be improved to facilitate access to physiotherapy and occupational therapy in SMA. Similarly, coverage of speech therapy in non-sitters and sitters, which can help provide more endurance and safe swallowing, was limited. Greater awareness of such symptoms (e.g. through proactive questioning) is recommended to ensure optimum supportive treatment [16, 20–23]. Similarly, with an overall prevalence of NIV of over 20%, we would also expect cough support to be used in all patients requiring NIV (at a minimum) [23]. Possible explanations for the identified gaps in rehabilitation care include lack of awareness of the need for rehabilitation treatment, limited availability of qualified therapists locally, difficulties in obtaining a prescription, or refusal by the patients themselves due to time constraints or low appreciation of the potential benefits [14]. Yet, this topic warrants further study to help improve prognosis and quality of life as better awareness of disease symptoms is needed to improve lung function and reduce respiratory impairment.

We found differences in relationship status of adults with SMA, where all walkers lived in marriage/relationship with their own family compared to sitters and non-sitters. In line with previous research, these findings suggests that motor skill impairment and disease comorbidity play a role in family planning [24–28].

Among adults with SMA, 60% had a high school diploma or other higher education entrance qualification and 36% had a university degree. These rates are almost twice as high as those derived for the German general population (34% and 18%, respectively) [29]. On the other hand, a significantly greater proportion of adults with SMA2 did not finish a vocational training (i.e., had not learned a profession) compared to SMA3. Although we cannot rule out that patients with academic education are more interested and willing to participate in research, this result could be an indication that the impact of the disease on motor functions at a young age encourages patients to opt for more academic education, as impairment of motor skills may discourage the choice of an apprenticeship.

The proportion of unemployed adults, 41% in all subgroups, was seven times higher than the German average (5.6%), which is somewhat surprising given their above-average education level [30]. Adults from the walker subgroup were less often unemployed than adults from the sitter and non-sitter groups, but still clearly above the German average. It seems reasonable to think that not only reduced motor function contributes to the high unemployment rate, but that the labour market generally is not sufficiently inclusive for people with chronic diseases, regardless of their vocational training. Therefore, future studies should investigate the reasons for the higher unemployment rate and support programs need to be developed to facilitate and improve vocational training and access to the labour-market for patients with SMA.

The main limitation of our study concerns external validity. Patients were recruited through a nationwide SMA registry for which participation is voluntary and patient/family-initiated. The response rate was only 21%. We therefore cannot rule out a degree of selection bias. Another limitation concerns information bias due to incorrect reporting. We tried to alleviate this problem by specifying recall periods in accordance with standard SMA care (based on our clinical experience and expertise of treating patients with SMA) and by including help texts, as well as logical tests and skip patterns to ensure that the collected data was accurate and complete. However, since patients also stated their type of SMA, this potential bias might also have impacted our comparison across SMA types.

## Conclusion

We show that the natural history of disease has changed in Germany following improvements in SMA care and the introduction of novel therapies. Yet, a non-trivial proportion of patients remain untreated. We also identified considerable limitations in rehabilitation and respiratory care, as well as low labour-market participation among adults with SMA, calling for action to improve the current situation.

## Methods

This was a cross-sectional, observational study of children and adults with SMA and one their caregivers in Germany. Study data was recorded directly from patient-caregiver pairs through a study questionnaire.

### Patient sample

Patients were identified and recruited trough the national SMA registry (www.sma-register.de) in Germany, part of the Translational Research in Europe-Assessment and Treatment of Neuromuscular Diseases (TREAT-NMD) network. TREAT-NMD was established in 2007 with the aim to facilitate identification and recruitment of patients to neuromuscular research, help disseminate best treatment practices, and raise awareness of limitations in current care. Participation in this registry is voluntary and free of charge. To be considered eligible to participate in this study, patients had to meet all of the following criteria: (1) genetically confirmed diagnosis of SMA, (2) ≥ 4 years of age, and (3) currently residing in Germany.

### Study procedures

An study invitation was sent via email by the SMA registry to all listed patients meeting the study criteria. Upon enrolment, patients and one of their caregivers were invited to complete a questionnaire administered online via a dedicated study website (www.soscisurvey.de) between June 14, and September 5, 2021. The study was approved by the regional Ethics Committee from the Saarland Medical Association on February 4, 2020, with protocol number 09/20 and registered at the German clinical trial register (DRKS00022876).

### Collected data

Participants were asked a series of questions about their demographic and clinical characteristics, including age, sex, SMA type, age at onset of first disease symptoms, age at diagnosis, current living situation, relationship status, highest level of education, highest level of vocational training, employment status, current best lower and upper extremity motor skills, current use of medical equipment for motor skills, scoliosis status, type of support with food intake and breathing, SMA specific medication, and current supportive therapy (questionnaire is available as Additional file 1).

Participants were classified based on SMA subtype (i.e., SMA1, 2, 3a, 3b and 4) as well as according to current best motor function of the lower limb and trunk (i.e., non-sitter, sitter, and walker). Children were defined as individuals younger than 18 years of age.

### Statistical analysis

All statistical analyses were performed with IBM SPSS Statistics version 28.0.1.1 (Inc., Chicago, IL). Continuous variables were described by means, standard deviations (SDs), and ranges. Categorical variables were described by absolute and relative frequencies. Within-group comparisons were performed with Analysis of variance (ANOVA) models for continuous variables and chi-square tests for categorical variables. A Bonferroni corrected *p* value of ≤ 0.016 was interpreted as statistically significant. Correlations were assessed using Pearson's test for dichotomous variables and Spearman's test for ordinal variables.

## Supplementary Information

**Additional file 1: **Questionnaire, English version. Questionnaire, original German version.

## Data Availability

The data that support the findings of this study are not publicly available due to ethical restrictions.

## Abbreviations

SMA: Spinal muscular atrophy
TREAT-NMD: Translational Research in Europe-Assessment and Treatment of Neuromuscular Diseases (TREAT-NMD) network: national SMA registry in Germany
SMN: Survival motor neuron
