# Supplementary material for: Areas of improvement in the medical care of SMA: evidence from a nationwide patient registry in Germany
Source: Orphanet J Rare Dis. 2023 Feb 21;18:32. doi: 10.1186/s13023-023-02639-z (PMC9945617; doi:10.1186/s13023-023-02639-z)
Supplement: Supplementary file 1 — Additional file 1: Questionnaire, English version. Questionnaire, original German version. [file 13023_2023_2639_MOESM1_ESM.docx]

**Supplementary material**

**Questionnaire, English version**

*Note from the author: This questionnaire was given to the participants in German.*

1. "Are you currently living in Germany?"

Options

1 = Yes

2 = No

2. “How old are you?"

Open input, whole number

**3.** "My gender is..."

Options

1 = female

2 = male

3 = diverse

**4.** "In which accommodation do you currently live?"

Options

1 = alone

2 = with my partner and possibly with children

3 = with my parents/one of my parents

4 = with other relatives

5 = foster family

6 = shared flat

7 = Wohngruppe (*Note from author: group home, form of less intensive residential care in Germany)*

8 = betreutes Wohnen (*Note from author: assisted living, form of less intensive residential care in Germany*)

9 = residential care home

10 = other accommodation

**5.** “What is your current marital status? (Multiple answers possible)"

Multiple choice

single

in a relationship

married

divorced

widowed

single parent

**6.** " What is the highest educational qualification you have obtained so far?"

Options

1 = no school-leaving qualification

2 = Hauptschlussabschluss (*Note from author: Certificate of Secondary Education, form of German school-leaving certificate after nine or ten years of school that qualifies to start an apprenticeship)*

3 = mittlere Reife (*Note from author: Certificate of Secondary Education, form of German school-leaving certificate after ten years of school that qualify to attend secondary school)*

4 = Fachabitur: (*Note from author: form of German vocational baccalaureate, a higher education qualification that entitles to study certain subjects and specialisations at universities and all subjects at universities of applied sciences)*

5 = Abitur (*Note from author: highest school-leaving qualification in Germany, a general higher education entrance qualification)*

**7.** "Which type of school did you attend? (Multiple selection possible)"

Multiple choice

Grundschule (*Note from author: German primary school)*

Hauptschule (*Note from author: form of German secondary school)*

Realschule (*Note from author: form of German secondary school)*

Gesamtschule (*Note from author: form of German secondary school with the possibility of obtaining the general higher education entrance qualification)*

Gemeinschaftsschule (*Note from author: form of German secondary school with the possibility of obtaining the general higher education entrance qualification)*

Gymnasium (*Note from author: form of German secondary school with the possibility of obtaining the general higher education entrance qualification)*

Förderschule (*Note from author: special school)*

Nicht beschult (*Note from author: no school attendance)*

other

**8.** "Have you previously completed vocational training? (Multiple answers possible)"

Multiple choice

without completed vocational training

completed vocational training/vocational school

completed studies at university or college

**9.** "What is your current employment status?"

Options

1 = employed

2 = unemployed

3 = self-employed

4 = not employed due to illness/disability/inability to work

5 = not employed due to child education/care of relatives

6 = in training/study/school

7 = retired

**10.** "What is the current employment status of your first parent?"

Options

1 = employed

2 = unemployed

3 = self-employed

4 = not employed due to illness/disability/inability to work

5 = not employed due to education/care of dependents

6 = in training/study/school

7 = retired

8 = no information/other

**11.** "What is the current employment status of your other parent?"

Options

1 = employed

2 = unemployed

3 = self-employed

4 = not employed due to illness/disability/inability to work

5 = not employed due to education/care of dependents

6 = in training/study/school

7 = retired

8 = no information/other

**12.** "The first symptoms of spinal muscular atrophy appeared when I was…”

... years old.

Open input, whole number

**13.** "When I was diagnosed with spinal muscular atrophy, I was .... years old."

Open input, whole number

**14.** " What is your diagnosed SMA subtype?"

Options

1 = Type I (onset of symptoms at the age of <6 months, no free sitting possible without therapy)

2 = Type II (onset of symptoms between 6 months and 2 years of age, usually able to sit without therapy but unable to walk freely).

3 = Type III (onset of symptoms until adolescence, possible loss of ability to walk)

4 = Type IV (onset of symptoms in adulthood)

5 = unknown

**15.** "What is currently your best motor skill?"

Options

1 = climbing stairs

2 = walking freely

3 = walking with assistive devices

4 = standing free

5 = standing with assistive devices

6 = sitting freely

7 = sitting with assistive devices

8 = none of the above

**16.** "Please tick what applies to you: (multiple selection possible)".

Multiple choice

I can...

hold a pencil or pick up a coin from the table with my fingers

raise my hands to my mouth while sitting

raise my hands above my head while sitting

none of the above

**17.** "Which of these assistive devices do you currently use in everyday life? (Multiple selection possible)"

Multiple choice

wheelchair

crutches or rollator

orthoses

none of these

**18.** "Do you currently require assistance with food intake?"

Options

1 = none

2 = I can´t chew well, but can eat soft/pureed food independently

3 = naso-gastric tube

4 = PEG tube (stomach tube through the abdominal wall)

5 = other support

**19.** "Have you been diagnosed with scoliosis (spinal curvature) to date?"

Options

1 = yes

2 = no

**20**. "Has your scoliosis (spinal curvature) been operated on so far?"

Options

1 = yes

2 = no

**21.** "Do you currently require respiratory support?"

Options

1 = no

2 = yes, cough assist

3 = yes, non-invasive ventilation

4 = yes, invasive ventilation (e.g. via a tracheostomy)

5 = yes, other respiratory support

**22.** "Have you received specific medication for SMA so far? (Multiple choice possible)"

Multiple choice

no

yes, Nusinersen (Spinraza®)

yes, Onasemnogen-Abeparvovec (Zolgensma®)

yes, Risdiplam (Evrysdi®)

**23.** "How long have you been receiving Nusinersen (Spinraza®)?”

since ... years

and ... months

Open input, whole numbers

“I no longer receive Nusinersen (Spinraza®)”

1 = not selected

2 = selected

**24.** "Are you currently using supportive therapies (physiotherapy, speech therapy, occupational therapy, etc.)? (multiple selection possible)"

Multiple selection

no

yes, physiotherapy.

yes, occupational therapy.

yes, speech therapy.

yes, other supportive therapies

**Questionnaire, original German version**

1. "Leben Sie aktuell in Deutschland?"

Auswahl

1 = Ja

2 = Nein

**2.** "Wie alt sind Sie?"

offene Eingabe, ganze Zahl

**3.** "Mein Geschlecht ist..."

Auswahl

1 = weiblich

2 = männlich

3 = divers

**4.** "In welcher Unterkunft leben Sie zur Zeit?"

Auswahl

1 = allein

2 = mit meinem Partner/meiner Partnerin und evtl. mit Kindern

3 = bei meinen Eltern/einem Elternteil

4 = bei anderen Verwandten

5 = Pflegefamilie

6 = Wohngemeinschaft

7 = Wohngruppe

8 = betreutes Wohnen

9 = Pflegeheim

10 = sonstige Unterkunft

**5.** "Was ist Ihr aktueller Familienstand? (Mehrfachnennung möglich)"

Mehrfachauswahl

single

in einer Beziehung

verheiratet

geschieden

verwitwet

alleinerziehend

**6.** "Was ist der höchste Bildungsabschluss, den Sie bisher erworben haben?"

Auswahl

1 = kein Schulabschluss

2 = Hauptschlussabschluss

3 = mittlere Reife

4 = Fachabitur

5 = Abitur

**7.** "Welche Schulform haben Sie besucht? (Mehrfachauswahl möglich)"

Mehrfachauswahl

Grundschule

Hauptschule

Realschule

Gesamtschule

Gemeinschaftsschule

Gymnasium

Förderschule

nicht beschult

sonstige

**8.** "Haben Sie bisher eine abgeschlossene berufliche Ausbildung? (Mehrfachnennung möglich)"

Mehrfachauswahl

ohne abgeschlossene Berufsausbildung

abgeschlossene Ausbildung/Berufsschule

abgeschlossenes Studium an Universität oder Hochschule

**9.** "Welchen Erwerbsstatus haben Sie aktuell?"

Auswahl

1 = angestellt

2 = arbeitslos

3 = selbstständig

4 = nicht erwerbstätig wegen Krankheit/Behinderung/Arbeitsunfähigkeit

5 = nicht erwerbstätig wegen Erziehung/Pflege von Angehörigen

6 = in Ausbildung/Studium/Schule

7 = berentet

**10.** "Welchen Erwerbsstatus hat Ihr erstes Elternteil aktuell?"

Auswahl

1 = angestellt

2 = arbeitslos

3 = selbstständig

4 = nicht erwerbstätig wegen Krankheit/Behinderung/Arbeitsunfähigkeit

5 = nicht erwerbstätig wegen Erziehung/Pflege von Angehörigen

6 = in Ausbildung/Studium/Schule

7 = berentet

8 = keine Angabe/sonstiges

**11.** "Welchen Erwerbsstatus hat Ihr anderes Elternteil aktuell?"

Auswahl

1 = angestellt

2 = arbeitslos

3 = selbstständig

4 = nicht erwerbstätig wegen Krankheit/Behinderung/Arbeitsunfähigkeit

5 = nicht erwerbstätig wegen Erziehung/Pflege von Angehörigen

6 = in Ausbildung/Studium/Schule

7 = berentet

8 = keine Angabe/sonstiges

**12.** "Die ersten Symptome von Spinaler Muskelatrophie traten bei mir auf, als ich"

... Jahre alt war.

Offene Eingabe, ganze Zahl

**13.** "Als bei mir die Diagnose Spinale Muskelatrophie gestellt wurde, war ich ... Jahre alt.“

Offene Eingabe, ganze Zahl

**14.** "Welcher SMA-Subtyp ist bei Ihnen diagnostiziert?"

Auswahl

1 = Typ I (Symptombeginn im Alter von <6 Monaten, ohne Therapie kein freies Sitzen möglich)

2 = Typ II (Symptombeginn im Alter von 6 Monaten bis 2 Jahren, in der Regel ohne Therapie Sitzfähigkeit aber kein

freies Gehen)

3 = Typ III (Symptombeginn bis zum Jugendalter, evtl. Verlust der Gehfähigkeit)

4 = Typ IV (Symptombeginn im Erwachsenenalter)

5 = unbekannt

**15.** "Was ist aktuell Ihre beste motorische Fähigkeit?"

Auswahl

1 = Treppen steigen

2 = freies Gehen

3 = Gehen mit Hilfsmitteln

4 = freies Stehen

5 = Stehen mit Hilfsmitteln

6 = freies Sitzen

7 = Sitzen mit Hilfsmitteln

8 = nichts davon

**16.** "Kreuzen Sie bitte an, was bei Ihnen zutrifft: (Mehrfachauswahl möglich)"

Mehrfachauswahl

Ich kann…

mit meinen Fingern einen Stift halten oder eine Münze vom Tisch aufheben

im Sitzen meine Hände zum Mund heben

im Sitzen meine Hände über meinen Kopf heben

nichts davon

**17.** "Welche dieser Hilfsmittel nutzen Sie aktuell im Alltag? (Mehrfachauswahl möglich)"

Mehrfachauswahl

Rollstuhl

Gehstützen oder Rollator

Orthesen

keine davon

**18.** "Benötigen Sie zur Zeit Unterstützung bei der Nahrungsaufnahme?"

Auswahl

1 = keine

2 = ich kann nicht gut kauen, aber weiches/püriertes Essen selbstständig essen

3 = Magensonde

4 = PEG-Sonde (Magensonde durch die Bauchdecke)

5 = sonstige Unterstützung

-9 = nicht beantwortet

**19.** "Wurde bei Ihnen bis zum jetzigen Zeitpunkt eine Skoliose (Wirbelsäulenverkrümmung) festgestellt?"

Auswahl

1 = ja

2 = nein

**20.** "Wurde Ihre Skoliose (Wirbelsäulenverkrümmung) bisher operiert?"

Auswahl

1 = ja

2 = nein

**21.** "Benötigen Sie aktuell atemunterstützende Maßnahmen?"

Auswahl

1 = nein

2 = ja, Hustenassistent

3 = ja, nicht-invasive Beatmung

4 = ja, invasive Beatmung (z.B. über ein Tracheostoma)

5 = ja, sonstige Atemunterstützung

**22.** "Haben Sie bis jetzt spezifische Medikamente für SMA bekommen? (Mehrfachauswahl möglich)"

Mehrfachauswahl

nein

ja, Nusinersen (Spinraza®)

ja, Onasemnogen-Abeparvovec (Zolgensma®)

ja, Risdiplam (Evrysdi®)

**23.** "Seit wann bekommen Sie Nusinersen (Spinraza®)?"

seit ... Jahren

und ... Monaten

Offene Eingabe, ganze Zahl

Ich bekomme kein Nusinersen (Spinraza®) mehr.

1 = nicht gewählt

2 = ausgewählt

**24.** "Nehmen Sie zur Zeit unterstützende Therapien (Physiotherapie, Logopädie, Ergotherapie etc.) in Anspruch? (Mehrfachauswahl möglich)"

Mehrfachauswahl

nein

ja, Physiotherapie.

ja, Ergotherapie.

ja, Logopädie.

ja, sonstige unterstützende Therapien.
